# Presurgical time and associated factors as predictors of acute perforated appendicitis: a prospective cohort study in a teaching pediatric hospital in Colombia

**DOI:** 10.1186/s12887-022-03121-8

**Published:** 2022-01-20

**Authors:** Paula Castro, Julián Rincón, Cristian Sánchez, Iván Molina, Giancarlo Buitrago

**Affiliations:** 1 Especialidad de Cirugía Pediátrica, Departamento de Cirugía, Universidad Nacional de Colombia, Calle 75a#102-25, Bogotá, 111321 Colombia; 2 Fundación Hospital Pediátrico La Misericordia, Bogotá, 111411 Colombia; 3 Facultad de Medicina, Universidad Nacional de Colombia, Universidad Nacional de Colombia, Bogotá, 111321 Colombia; 4 Hospital Universitario Nacional de Colombia, Bogotá, 111321 Colombia

**Keywords:** Appendicitis, Pediatric, Presurgical time, Out-of-hospital, Risk factors

## Abstract

**Background:**

We aim to determine the association between out and in-hospital factors with time, from the beginning of the symptoms to the surgery, in patients with acute appendicitis treated at Fundación Hospital Pediatrico La Misericordia (HOMI) in Colombia.

**Methods:**

Eleven month prospective cohort study of pediatric patients at HOMI with acute appendicitis diagnosis taken to surgery. Data from the out-of-hospital phase was collected by surveying parents, and the data regarding the in-hospital phase was completed with medical records. We analyzed the association between the time from the beginning of the symptoms to the surgery, and out and in-hospital factors associated with this time using generalized linear models.

**Results:**

Eight hundred three patients were included in the study. Total pre-surgical time was longer in perforated appendicitis (PA) group (2.65 days, standard deviation (SD) 1.88 vs. 2.04 days, SD 1.45) (*p* < 0.01). Factors associated with longer total and out-of-hospital presurgical times were age under 4 years old, lower socioeconomic status, father as a caregiver, self-medication, and underestimating disease severity.

**Conclusions:**

Out-of-hospital timing determines the longer pre-surgical time in complicated appendicitis. Younger age and lower socioeconomic status affect time significantly. We suggest the implementation of strategies in order to lower prehospital time, rates, and costs of complicated appendicitis.

**Supplementary Information:**

The online version contains supplementary material available at 10.1186/s12887-022-03121-8.

## Background

Acute appendicitis is the most common surgical cause of abdominal pain in children [1]. Different cohorts report an incidence of pediatric acute appendicitis between 1.13 to 1.39 per 1000 per year [2], being less frequent in children under 4 years [3]. In 2017, pediatric surgeons performed 1450 appendectomies at Fundación Hospital Pediatrico La Misericordia (HOMI) in Bogotá.

In children, atypical or nonspecific symptoms and variable disease course, especially in neonates and children under 1 year of age, contribute to an early appendicular obstruction and late presentation [4–6]. Additionally, less developed omentum and less intra-abdominal fat facilitate the spread of peritonitis [4–6]. In worldwide literature, perforation frequency is higher in children than adults, with incidences between 15 and 50% [1, 7, 8]. Patient age is tied closely to the stage of acute appendicitis, so the youngest patients present with more advanced stages of the disease and are at greater risk of perforation, with a recent study showing a significant increase of perforation in relation to age as follows: 100% < 1 year; 100% 1–2 years; 83.3% 2–3 years; 71.4% 3–4 years; 78.6% 4–5 years and 47.3% - 5 years [9]. In Colombia, a single national report shows 25.8% of appendectomies with peritoneal drainage [7]. At our institution, we found a 41.3% perforation rate in acute appendicitis in 2017.

Perforated appendicitis (PA) is associated with higher postoperative morbidity and mortality, prolonged hospital stays, and higher costs [1, 7, 10]. There are no comparative studies in our country [7].

A linear increase in perforation percentage after 24 h of the onset of symptoms has been described [1, 6, 11]. When analyzing the duration of symptoms before admission to the hospital, a more significant delay in the out-of-hospital phase was found in patients with PA compared to non-perforated appendicitis (NPA) group, which is a factor that predicts perforation [10, 12, 13]. In cohorts carried out in developed countries, race and socioeconomic status are factors described associated with late consultation, which are preventable if access to health service is guaranteed [13–16]. Nevertheless, there are no reports in the literature that assess those demographic factors and their influence on presurgical time in low-income and medium-income countries.

Concerning in-hospital timing, [10, 17] there is no impact on the 30-day morbidity rate if appendectomy is carried out between 6 and 12 h after the patient’s admission [10, 17]. But performing a late surgery (more than 48 h after admission) is associated with a higher probability of complications and a more extended hospital stay [18]. Several paraclinical indicators and scoring systems have been described as predictors of the probability of acute PA. The Alvarado score is a frequently utilized tool for acute appendicitis symptom gradation, yet it lacks specificity and sensitivity [19]. Recently, Appendicitis inflammatory score (AIR) has been created to overcome shortcomings of the Alvarado score and Pediatric appendicitis score [20]. Recent studies validated the AIR score and reported that the AIR score significantly outperforms the older Alvarado score, especially in distinguishing simple from advanced appendicitis but still not enough to be exclusive criteria in establishing the diagnosis of acute appendicitis [21]. However, it has not been analyzed if the presence of the factors evaluated in these scores determines a shorter or longer time of preoperative hospital stay in patients with acute PA [4, 12].

In a country where health access is limited in several areas, factors associated with higher out-of-hospital time could be distinct or impact in a different magnitude than the observed in developed countries. This is the first study in our setting regarding the time from the onset of symptoms to surgical management of pediatric appendicitis. We aim to analyze the association between out-of-hospital and in-hospital factors with the time elapsed before and after admission to our institution and determine its impact in the presence of perforation in acute appendicitis.

## Methods

### Type of study

We conducted a prospective cohort study of children under 17 years old diagnosed with acute appendicitis treated at HOMI between January 10th, 2018, and December 16th, 2018. HOMI is a high-level teaching pediatric hospital located in the city of Bogotá with 336 beds. It is a reference hospital; therefore, a population from all over the country is transported there to receive health care. The department of pediatric surgery provides care of patients with acute abdominal pain and after the clinical diagnosis of acute appendicitis, the institutional protocol indicates the performance of a laparoscopic appendectomy within the next 8 h. Sixteen pediatric surgeons participated in this study, with at least 5 years of experience acquired in pediatric surgery formation, and approximately 50 patients were operated on by each surgeon.

During the study period, all caregivers of children with a preoperative clinical or image-based diagnosis of acute appendicitis were asked to participate and informed consent was signed for this purpose. Once acute appendicitis was diagnosed, an antibiotic was prescribed according to the institutional protocol: ampicillin-sulbactam or piperacillin-tazobactam in cases with suspicion of generalized peritonitis. All included children were followed up to the completion of hospitalization and the first postoperative control, which was performed between 15 and 21 days after the surgery. This study was approved by the HOMI research ethics committee (Record number 005, CEI 49–17, August 25, 2017).

### Exposure and covariates

The primary exposure variable was the total pre-surgical time, which was defined as the time elapsed from the onset of symptoms to surgery. This time was divided between out-of-hospital pre-surgical time and in-hospital pre-surgical time. The first was defined as the time between the onset of symptoms and admission to HOMI, and the second, as the time from admission to operation. For this, a survey was carried out to the caregiver, which contained questions that allowed to approximate the day and time of onset of symptoms, defined as the moment when at least one of the following symptoms appears: abdominal pain, vomiting, anorexia, diarrhea, or fever. The date and time of arrival at HOMI and the surgery were obtained from the hospital’s electronic medical record. Additionally, we collected information concerning characteristics of the patient, their guardians, or situations before the hospital arrival: age of the child, caregiver (mother, father, or other); caregiver’s age, education, and employment status; residence (rural or urban); self-medication, home management of abdominal pain; causes of delay for a first out-patient visit; conduct at the first out-patient visit and number of out-patient visits before admission to our institution. In addition to the out-of-hospital variables, other clinical covariates of the child were measured at the time of admission and included: overall status, pain, heart rate, respiratory rate, temperature, and results of paraclinical examinations.

### Outcomes

The diagnosis of PA was established if one or more of the following criteria was present: the spread of pus with the visible appendicolith in the abdominal cavity or visible perforation of the appendix [22]. PA was defined only by intraoperative findings because in our institution we have identified adequate concordance between surgical and histopathological PA diagnosis with no significant difference in the postoperative course if only microscopic perforation is reported by the pathologist [23]. After establishing the presence of acute PA, the primary endpoint of interest of this study was to determine the factors associated with out-of-hospital and in-hospital presurgical time in PA.

### Statistical analysis

Descriptive analysis of all variables in the cohort and bivariate analyses between the baseline characteristics and the presence or absence of perforation was performed.

Continuous variables were compared with T-tests and categorical variables with chi2 tests. Multivariate analysis with generalized linear models was used to identify associations between exposures and outcomes of interest, controlling for possible observable confounders.

These analyses were carried out for two purposes: i. To ascertain the association between pre-surgical times and the incidence of PA; and ii. Determine the prehospital factors (sociodemographic, symptoms, home management, and 1st out-patient visit conduct) associated with preoperative times. For the first one, multivariate logistic regression was performed in which the dependent variable was the presence or absence of perforation as a function of the pre-surgical times, controlling some clinical and sociodemographic characteristics. For the second analysis, multiple linear regressions were performed where the dependent variables were the total pre-surgical time and the out-of-hospital pre-surgical time, based on all possible associated factors. In both multivariate analyzes, variables to be included were selected based on statistical significance and based on clinical significance. Statistical significance was identified from differences in the distribution of each variable in relation to the outcome (univariate analysis). Clinical significance was identified from previous literature and expert recommendation. All estimators were calculated with 95% confidence intervals, *p*-values lower than 0.05 were considered as significant and analyses were performed in Stata 14® (MP—Parallel Edition, StataCorp LLC, College Station, Texas).

## Results

### Cohort characteristics

From January to December 2018, 803 patients with acute appendicitis were included. Forty patients were excluded because of a lack of caregiver’s consent. The mean age was 10.7 years (Standard Deviation –SD- 3.89), with a male predominance (60.7%). 60.3% of the patients had acute perforated appendicitis. The rest of the information corresponding to sociodemographic and clinical characteristics of the patients and caregivers at the time of admission to our institution is listed in Table 1. Variables before admission and at admission are reported in Additional file 1. Paraclinical variables were not significantly different between subgroups.

**Table 1:** Sociodemographic characteristics

Patient characteristics	Full sample	Non-perforated appendicitis	Perforated appendicitis	***p***-value
All patients, No. (%)	803 (100)	319 (39.73)	484 (60.27)	
Age, mean (SD), years	10.692 (3.898)	11.295 (0.201)	10.295 (0.184)	**0.000**
Age categories, N (%), years				
0–4 years	66 (8.20)	12 (18.18)	54 (12.7)	**0.000**
5–9 years	273 (34.00)	105 (38.46)	168 (34.71)	
10–17 years	464 (57.78)	202 (43.53)	262 (54.13)	
Gender, No. (%)				
Female	315 (39.23)	109 (34.17)	206 (42.56)	**0.017**
Male	488 (60.77)	210 (65.83)	278 (57.44)	
Domicile, No. (%)				
Rural	94 (11.71)	31 (9.72)	63 (13.02)	0.155
Urban	709 (88.29)	288 (90.28)	421 (86.98)	
Socioeconomic status, No (%)				
1: very low	149 (18.56)	47 (14.73)	102 (21.07)	0.072
2: low	426 (53.05)	175 (54.86)	251 (51.86)	
3: intermediate	228 (28.39)	97 (30.41)	131 (27.07)	
**Caregiver characteristics**				
Caregiver, No. (%)				
Mother	665 (82.81)	260 (81.50)	405 (83.68)	0.720
Father	113 (14.07)	48 (15.50)	65 (13.43)	
Other	25 (3.11)	11 (3.45)	14 (2.89)	
Age mean (SD), years	35.73 (8.03)	36.23 (7.89)	35.40 (8.11)	
Gender (%)				
Female	688 (85.67)	270 (84.64)	418 (86.36)	0.495
Male	115 (14.32)	49 (15.36)	66 (13.64)	
Current worker (%)				
No	179 (22.29)	62 (19.44)	117 (24.17)	0.114
Yes	624 (77.71)	257 (80.56)	367 (75.83)	
Education level, title (%)				
Elementary school or less	130 (16.19)	43 (13.48)	87 (17.98)	0.166
High school	357 (44.45)	141 (44.20)	216 (44.63)	
Tertiary education^a^	316 (39.35)	135 (42.32)	181 (37.40)	

^a^Doctorate, Professional, Bachelor’s or Master’s degree

*SD* standard deviation

### Time comparison between perforated and non-perforated appendicitis

**Fig. 1:**
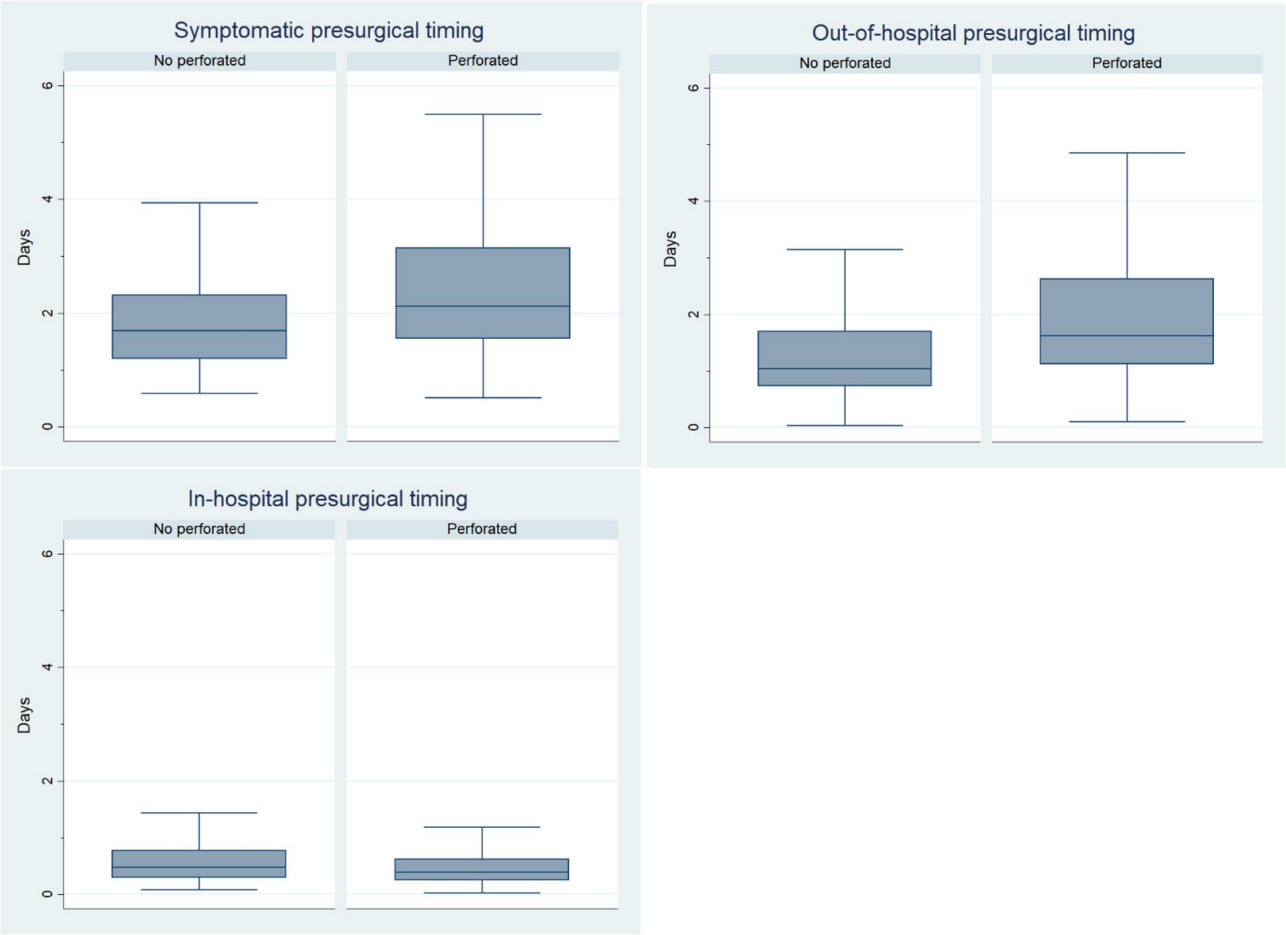
Distribution of presurgical times between patients with perforated and non-perforated appendicitis

We analyzed total pre-surgical time, out-of-hospital pre-surgical time, and in-hospital presurgical time. Figure 1 and Table 2 show the measured times comparing NPA and PA groups. We found statistically significant differences between the two groups in the total symptomatic time and total pre-surgical time was shorter in the NPA group (Mean: 2.04 [SD 1.45] vs. 2.65 [SD 1.88] days, *p* < 0.01).

**Table 2:** Distribution of presurgical timing for perforated and non-perforated acute appendicitis

Presurgical timing	Mean (days)	SD	p25^a^	p50^b^	p75^c^	Min	Max
Total presurgical timing							
Non perforated	2.046*	1.451	1.194	1.687	2.317	0.590	13.323
Perforated	2.655*	1.887	1.550	2.115	3.149	0.507	14.334
Total	2.413	1.751	1.396	1.951	2.799	0.507	14.334
Out-of-hospital presurgical timing							
Non perforated	1.457*	1.375	0.727	1.044	1.696	0.042	12.723
Perforated	2.154*	1.846	1.115	1.626	2.621	0.102	14.130
Total	1.877	1.709	0.889	1.412	2.324	0.042	14.130
In-hospital presurgical timing							
Non perforated	0.589*	0.419	0.294	0.484	0.774	0.082	2.685
Perforated	0.501*	0.425	0.246	0.391	0.624	0.026	4.609
Total	0.536	0.425	0.259	0.411	0.687	0.026	4.609

*p < 0.01

*SD* standard deviation

^a^ p25 percentile 25th, ^b^ p50 percentile 50th, ^c^ p75 percentile 75th,

Table 3 show multivariate logistic regressions between presurgical times as a predictor of PA including the following control variables: age, gender, caregiver’s age, employment status and education, socioeconomic status, pain, heart and respiratory rate, temperature, leukocyte and neutrophil count at admission. Model 1 shows the positive association between longer presurgical total time with the event of perforation (odds ratio [OR], 1.34; 95% CI (confidence interval), 1.18–1.54; *p* < 0.01). When subdivided in categories the association increased as the total presurgical time was longer compared with 0–1 day (OR, 2.14; 95%CI,1.43–3.20 for 2 days, *p* = .00 Vs OR,3.61; 95%CI,1.59–8.21 for 5 or more days, p = .00) (Model 2). In the adjusted model 3, odds of perforation were higher in the analysis regarding out-of-hospital presurgical timing (OR, 1.39; 95% CI, 1.21–1.61; p = .00), however, the association was not statistically significant concerning in-hospital pre-surgical timing.

**Table 3:** Association between presurgical timing and rate of perforation

	Model 1				Model 2				Model 3			
	OR	*p*-value	[95% Conf.	Interval]	OR	*p*-value	[95% Conf.	Interval]	OR	*p*-value	[95% Conf.	Interval]
Total presurgical timing												
Continuous variable	1.34	0.00	[1.18	1.53]								
Categories												
0–1 day					1.00	NA						
2 days					2.14	0.00	[1.43	3.20]				
3 days					3.16	0.00	[1.78	5.63]				
4 days					2.61	0.02	[1.18	5.81]				
5 or more days					3.61	0.00	[1.59	8.21]				
Out-of-hospital presurgical timing									1.39	0.00	[1.21	1.61]
In-hospital presurgical timing									0.98	0.91	[0.67	1.44]

*Model 1.* Exposure variable: total pre-surgical symptomatic time (days)

*Model 2*. Exposure variable: total pre-surgical symptomatic time (5 categories)

*Model 3*. Exposure variable: Out-of-hospital and in-hospital presurgical time

### Factors associated with time

Table 4 shows the multivariate linear regression between total pre-surgical time and out-of-hospital pre-surgical time with several factors: sociodemographic, home management, clinical, and out-hospital visit conduct.

**Table 4:** Factors associated with presurgical and out-of-hospital timing in complicated appendicitis

Factors	Total pre-surgical timing (days)		Out-of-hospital presurgical timing (days)	
	Multivariate analysis		Multivariate analysis	
	Coef (95% CI)	*p* value	Coef (95% CI)	*p* value
Age				
0–4 years	0.69 (0.26–1.13)	.00	0.68 (0.26–1.1)	.00
5–9 years	0.15 (−0.10–0.39)	.24	0.20 (−0.02–0.44)	.085
≥ 10 years	Ref			
Gender				
Male	0.08 (−0.31–0.14)	.48	−0.01 (− 0.24–0.2)	.87
Domicile				
Urban	−0.29 (− 0.649–0.066)	.11	− 0.26 (− 0.69- -0.02)	.12
Socioeconomic status				
2	− 0.44 (− 0.75- -0.13)	.01	− 0.46 (− 0.76- -0.16)	.00
3	− 0.34 (− 0.69–0.01)	.06	−0.36 (− 0.69- -0.02)	.036
1	Ref			
Caregiver				
Father	0.38 (0.05–0.70)	.02	0.41 (0.10–0.73)	.01
Other	−0.13 (−0.799–0.529)	.69	−1.89 (− 0.82–0.44)	.56
Age				
25–39 years	−0.16 (− 0.72–0.39)	.55	´-.30 (−83- -.22)	.26
> 40 years	−0.15 (− 0.73–0.44)	.62	´-.31 (−.88–.24)	.27
≤ 24 years	Ref			
Education				
High school	0.12 (−0.21–0.45)	.47	0.02 (−0.29- -0.33)	.90
Tertiary degree	0.03 (−0.32–0.38)	.876	− 0.11 (− 0.44–0.22)	.52
Elementary school or less	Ref			
Selfmedication				
Yes	0.29 (0.05–0.52)	.02	0.33 (0.11–0.56)	.00
Home management				
Herbal hot water	0–0.16 (−0.42–0.12)	.26	−0.17 (− 0.43–0.08)	.19
Massage	0.04 (− 0.31–0.39)	.82	0.02 (− 0.31–0.40)	.91
Other	0.06 (− 0.32–0.43)	.75	0.04 (− 0.31–0.40)	.80
None	Ref			
Delay 1st out-patient visit (cause)				
Didn’t believe it was serious	0.54 (− 0.19–0.89)	.00	.608 (.27–.94)	.00
Other	0.41 (− 0.02–0.84)	.06	.42 (.01–.83)	.05
1st out-patient visit conduct				
Discharge	1.61 (1.2–1.95)	.00	1.68 (1.3–2.0)	.00
Referal	−0.02 (− 0.27–0.23)	.88	0.05 (− 0.18–0.28)	.67

Age between 0 and 4 years, low socioeconomic status, father as the caregiver, self-medication, underestimating the severity of the disease for the first out-patient visit, and medical discharge in the first out-patient visit were factors significantly associated with longer total and out-of-hospital presurgical times.

## Discussion

This is the first prospective study in the Colombian pediatric population that analyzes pre-surgical time and out-of-hospital factors associated with PA. During the 11 months of the study, 803 patients with acute appendicitis were included. We found statistically significant differences between gender (predominantly male) and slightly younger age in the PA group [13, 17]. 60.3% of our cohort had higher acute PA incidence than previously reported [7]. 11.7% of the patients came from rural areas, and 71.6% had low socioeconomic status. Although there were no statistically significant differences between the two groups, in our context, these factors could be related to limited access to health care, which has been associated with higher rates of PA [16]. The results from this study found longer time lapses in the PA group regarding total pre-surgical time than in other cohorts [8, 23]. There was a difference in pre-surgical time of 0.65 days among the PA and NPA groups (Table 2), which adjusted in the multivariate logistic regressions showed increased odds of PA as the time was longer (time blocks), lower than previously reported [8, 17].

When distinguishing between out-of-hospital and in-hospital time, the PA group showed a mean out-of-hospital time of 2.15 days compared to 1.45 days in the NPA group. As reported by Cameron et al., our PA group also presented less in-hospital time, probably because our institution is a pediatric hospital and additionally because the pediatric surgery team prioritizes surgical intervention in this group of patients [24]. Our investigation confirmed that perforation is associated with an out-of-hospital delay in our population rather than in-hospital delay, analogous to literature in developed countries [8, 24, 25].

Regarding demographic and socioeconomic factors for delayed appendectomies, children in the PA group had lower socioeconomic status, but interestingly not significant association with educational level, employment status, or residence [26]. Additionally, concerning complex factors that determine health care utilization, we do not rule out the limitation of economic resources as a cause of underestimating the severity of the disease as previously reported by Baxter et al. [16]. Considering this last issue, in the Appendicitis Patient Pre-Hospital Experience (APPE) survey for caregivers, parents last to appear described reduced social support and a tendency to “wait it out” [27]. 64.3% of parents in our cohort of PA manifested underestimating disease severity compared with 35.7% in the NPA group, but we did not further inquire the exact cause.

Age less than 4 years was another factor related to longer pre-surgical time in the PA group. It has been previously described that patients with complicated appendicitis are younger than patients with non-complicated appendicitis, but this factor was not previously reported related to delay in time [8, 17].

Other factors associated with PA in the present study were the father being the primary caregiver and self-medication (64.1% vs. 35.8%). The authors did not find literature regarding caregivers in appendicitis; nevertheless, there are some reports in other conditions that describe female caregivers have better knowledge concerning child’s condition [28–33]. We consider self-medication is associated with complicated appendicitis because of the false improvement in response to analgesics.

Our study may be limited due to a recall bias common to this type of outcomes research as some of the data recollection was done by surveying the caregiver in the out-of-hospital pre-surgical time, data accuracy might be lower in the NPA. Furthermore, being a high-level referral hospital for childcare patients referred with acute appendicitis have already had previous consultations which may affect out-of-hospital time and PA incidence.

## Conclusions

Out-of-hospital timing determines the longer pre-surgical time in complicated appendicitis. Younger age and lower socioeconomic status affect time significantly. Problematic social disparities are difficult to assess, but we believe that education for the overall population, through general teaching campaigns including enhanced parent awareness of warning signs related to abdominal pain and standardized approaches to abdominal pain in first care facilities could lower prehospital time and, therefore, rates and costs of complicated appendicitis.

## Supplementary Information

**Additional file 1.** Before admission and Admission characteristics.

## Data Availability

The datasets generated and/or analyzed during the current study are not publicly available due to patient confidentiality (children) but are available from the corresponding author on reasonable request.

## Abbreviations

HOMI: Fundación Hospital Pediatrico La Misericordia
PA: Perforated appendicitis
SD: Standard deviation
NPA: Non-perforated appendicitis
AIR: Appendicitis inflammatory score
OR: Odds ratio
CI: Confidence interval
APPE: Appendicitis Patient Pre-Hospital Experience
