# Supplementary material for: Presurgical time and associated factors as predictors of acute perforated appendicitis: a prospective cohort study in a teaching pediatric hospital in Colombia
Source: BMC Pediatr. 2022 Jan 20;22:49. doi: 10.1186/s12887-022-03121-8 (PMC8772156; doi:10.1186/s12887-022-03121-8)
Supplement: Supplementary file 1 — Additional file 1. Before admission and Admission characteristics. [file 12887_2022_3121_MOESM1_ESM.docx]

**Additional file 1**. Before admission and Admission characteristics.

| **Variables before admission** | **Full sample** | **Non-perforated appendicitis** | **Perforated appendicitis** | **p-value** |
| --- | --- | --- | --- | --- |
| Home management (%) |  |  |  |  |
| Herbal hot water | 305 (37.98) | 117 (36.68) | 188 (38.84) | 0.185 |
| Massage | 129 (16.06) | 43 (13.48) | 86 (17.77) |  |
| Other | 103 (12.83) | 41 (12.85) | 62 (12.81) |  |
| None | 265 (33.13) | 118 (36.99) | 148 (30.58) |  |
| Self medication (%) |  |  |  |  |
| Yes | 458 (57.04) | 164 (51.41) | 294 (60.74) | **0.009** |
| No | 345 (42.96) | 155 (48.59) | 190 (39.26) |  |
| Cause of delay, No. (%) | | | | |
| Didn´t believe it was serious | 577 (71.86) | 206 (64.58) | 371 (76.65) | **0.000** |
| Other | 122 (15.19) | 54 (16.93) | 68 (14.05) |  |
| None | 104 (12.95) | 59 (18.50) | 45 (9.30) |  |
| Outpatient first visit, No. (%) |  |  |  |  |
| Additional studies | 320 (39.85) | 134 (42.01) | 186 (38.42) | **0.000** |
| Hospital discharge | 131 (16.31) | 25 (7.84) | 106 (21.9) |  |
| Remision | 352 (43.84) | 160 (50.16) | 192 (39.66) |  |
| Number of outpatient visits, No. (%) | | | | |
| 0 | 125 (15.57) | 56 (17.55) | 69 (14.25) | **0.000** |
| 1 | 508 (63.26) | 218 (68.34) | 290 (59.91) |  |
| >=2 | 170 (21.17) | 45 (14.11) | 125 (25.82) |  |
| **Variables at admission** | | | | |
| Overall status, No. (%) |  |  |  |  |
| Good | 615 (76.59) | 271 (84.95) | 344 (71.07) | **0.000** |
| Regular | 184 (22.91) | 48 (15.05) | 136 (28.10) |  |
| Bad | 4 (0.5) | 0 (0.00) | 4 (0.83) |  |
| Visual analogue scale for pain, No. (%) | | | |  |
| <=4 | 76 (9.46) | 41 (12.85) | 35 (7.23) | **0.000** |
| 6 | 145 (18.06) | 74 (23.20) | 71 (14.67) |  |
| 8 | 306 (38.11) | 148 (46.39) | 158 (32.64) |  |
| 10 | 276 (34.37) | 56 (17.55) | 220 (45.45) |  |
| Respiratory distress, No. (%) |  |  |  |  |
| No | 798 (99.38) | 317 (99.37) | 481 (99.38) | 0.659 |
| Mild | 1 (0.12) | 0 (0.00) | 1 (0.21) |  |
| Moderate | 4 (0.50) | 2 (0.63) | 2 (0.41) |  |
| Physical exam, No. (%) |  |  |  |  |
| No pain | 17 (2.12) | 9 (2.82) | 8 (1.65) | **0.000** |
| Mild pain | 106 (13.20) | 57 (17.87) | 49 (10.12) |  |
| Moderate pain | 161 (20.05) | 80 (25.08) | 81 (16.74) |  |
| Acute abdomen | 512 (63.76) | 172 (53.92) | 340 (70.25) |  |
| Difficult assessment | 7 (0.87) | 1 (0.91) | 6 (1.24) |  |
| Heart rate (SD) | 113.69 (22.14) | 104.76 (20.41) | 119.58 (21.26) |  |
| Respiratory rate (SD) | 22.65 (3.51) | 22.17 (2.86) | 22.96 (3.56) |  |
| Temperature (SD) | 37.09 (0.90) | 36.80 (0.73) | 37.28 (0.95) |  |
| WBC* count per uL (SD) | 15642 (5620) | 14470 (5102.38) | 16450 (5823.34) |  |
| Neutrophil count per uL (SD) | 12534 (5434) | 11165 (5047.97) | 13479.94 (54.98.13) |  |

*WBC: White Blood Cell.

*SD* standard deviation
